# A Molecularly Imprinted Polymer-based Dye Displacement Assay for the Rapid Visual Detection of Amphetamine in Urine

**DOI:** 10.3390/molecules25225222

**Published:** 2020-11-10

**Authors:** Joseph W. Lowdon, Kasper Eersels, Rocio Arreguin-Campos, Manlio Caldara, Benjamin Heidt, Renato Rogosic, Kathia L. Jimenez-Monroy, Thomas J. Cleij, Hanne Diliën, Bart van Grinsven

**Affiliations:** Sensor Engineering Group, Faculty of Science and Engineering, Maastricht University, PO Box 616, 6200 MD Maastricht, The Netherlands; kasper.eersels@maastrichtuniversity.nl (K.E.); r.arreguincampos@maastrichtuniversity.nl (R.A.-C.); m.caldara@maastrichtuniversity.nl (M.C.); benjamin.heidt@maastrichtuniversity.nl (B.H.); renato.rogosic@maastrichtuniversity.nl (R.R.); k.jimenezmonroy@maastrichtuniversity.nl (K.L.J.-M.); thomas.cleij@maastrichtuniversity.nl (T.J.C.); hanne.dilien@maastrichtuniversity.nl (H.D.); bart.vangrinsven@maastrichtuniversity.nl (B.v.G.)

**Keywords:** molecularly imprinted polymers, colorimetry, displacement assay, amphetamine

## Abstract

The rapid sensing of drug compounds has traditionally relied on antibodies, enzymes and electrochemical reactions. These technologies can frequently produce false positives/negatives and require specific conditions to operate. Akin to antibodies, molecularly imprinted polymers (MIPs) are a more robust synthetic alternative with the ability to bind a target molecule with an affinity comparable to that of its natural counterparts. With this in mind, the research presented in this article introduces a facile MIP-based dye displacement assay for the detection of (±) amphetamine in urine. The selective nature of MIPs coupled with a displaceable dye enables the resulting low-cost assay to rapidly produce a clear visual confirmation of a target’s presence, offering huge commercial potential. The following manuscript characterizes the proposed assay, drawing attention to various facets of the sensor design and optimization. To this end, synthesis of a MIP tailored towards amphetamine is described, scrutinizing the composition and selectivity (ibuprofen, naproxen, 2-methoxphenidine, quetiapine) of the reported synthetic receptor. Dye selection for the development of the displacement assay follows, proceeded by optimization of the displacement process by investigating the time taken and the amount of MIP powder required for optimum displacement. An optimized dose–response curve is then presented, introducing (±) amphetamine hydrochloride (0.01–1 mg mL^−1^) to the engineered sensor and determining the limit of detection (LoD). The research culminates in the assay being used for the analysis of spiked urine samples (amphetamine, ibuprofen, naproxen, 2-methoxphenidine, quetiapine, bupropion, pheniramine, bromopheniramine) and evaluating its potential as a low-cost, rapid and selective method of analysis.

## 1. Introduction

Discovered in 1887, amphetamine has notoriously been a drug of abuse over the decades, with its uses varying as a cognitive enhancer, athletics enhancer, treatment for nasal congestion, an antidepressant, a euphoriant and even as an aphrodisiac [1,2,3,4]. The name amphetamine commonly refers to both enantiomers being (D) dextroamphetamine and (L) levoamphetamine, with D-amphetamine exhibiting a more pronounced effect on the central nervous system (CNS) [5]. This activity and the list of potential uses poses an issue from both a medical and analytical standpoint, giving rise to various challenges that must be overcome. Confirmation of the presence of amphetamine in a host’s urine is an analytical minefield, with current rapid screening methods failing (e.g., immunoassays, ELISA) to discriminate between amphetamine and related compounds such as tricyclic compounds, antidepressants (quetiapine, bupropion), anti-inflammatory drugs (ibuprofen, naproxen) certain antihistamines (pheniramine, bromopheniramine), nasal inhalers and even some cold/flu medications [6]. Therefore, developing a test that can easily discriminate between this wide array of compounds is essential. The most prevalent methods of amphetamine detection are conducted on blood and/or urine [7]. Urine analysis has the advantage of being noninvasive and posing no risk to the patient. This removes the complexities and costs commonly associated with plasma analysis; therefore, the possibility of the detection of amphetamine in urine using molecularly imprinted polymers seems the most interesting route from a commercial point-of-view.

Molecularly imprinted polymers (MIPs) have been a rapidly developing field over the last couple of decades, demonstrating selective analyte binding affinities on par with those of enzymes [8,9,10,11,12]. In addition to being a valuable asset in separation sciences, catalysis and sensor applications [13,14,15,16,17,18,19,20,21,22,23,24,25,26,27,28,29], MIPs have been incorporated into a wide range of colorimetric assays over the years. The conversion of a MIP into a colorimetric tool has many approaches, with various molecular mechanisms being employed to achieve the desired colorimetric verification (quantum dots, dye displacement assays, enzymatic reactions) [30,31,32,33,34,35,36]. The most straightforward approach was introduced by McNiven et al. Chloramphenicol MIPs were loaded with a dye-labeled analog that was displaced when the dye-loaded MIP particles were incubated with the template [37]. Greene et al. demonstrated later that the displaced dye molecule does not have to share a perfect resemblance to the target molecule, opening up the possibility of using a wide range of other dye molecules [38]. Benzofurazan dyes were used as examples, generating a competitive binding assay to detect the presence of six imprinted amine compounds. The authors of this paper extended the concept recently, presenting the use of commercially available dyes (malachite green) in a MIP-based dye displacement assay for the detection of diarylethylamines that was coined substrate displacement colorimetry (SDC) [39].

**Figure 1:**
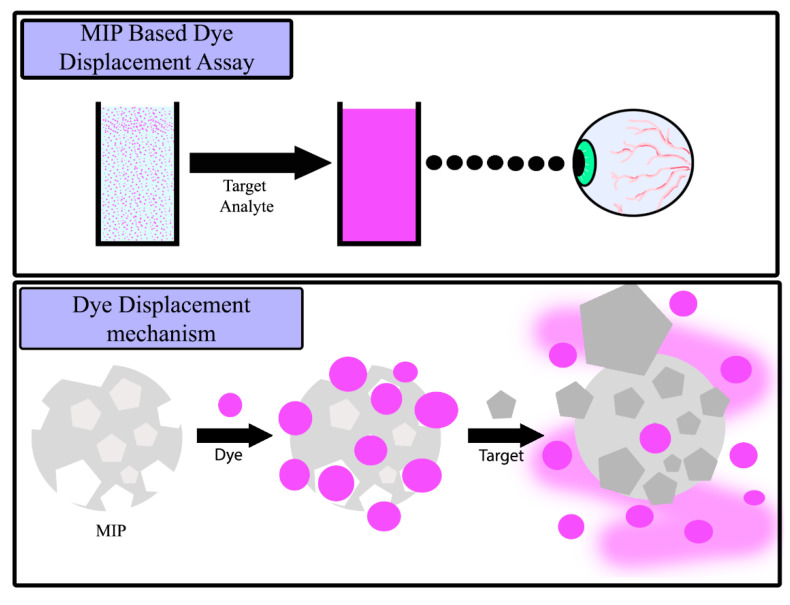
Artistic interpretation of observable color change when the molecularly imprinted polymer (MIP)-based dye displacement assay is exposed to a solution containing a target analyte, and an interpretation of the driving force behind the molecular mechanism.

In essence, SDC is a method of converting MIPs into a visual analytical tool by prebinding a dye molecule into the nanocavities available in the MIP. Upon the addition of the template molecule, the prebound dye molecule is released due to the higher affinity of the template molecule towards the complimentary MIP. This process therefore qualifies as a displacement reaction, as a molecule immobilized in the binding cavities gets displaced by the presence of a molecule with a higher affinity for the MIP through competitive binding. Compared to other implementations of MIPs into sensing arrays, this MIP-based dye displacement assay offers a fast and straightforward method of gaining rapid visual confirmation of a target analyte (Figure 1).

In this article, we investigate the possibility of applying this technique towards the detection of amphetamine, generating a rapid colorimetric test for its presence in urine. To this end, a MIP is developed towards the binding of amphetamine and its selectivity is demonstrated in the presence of common molecular interferences. A full study on the binding of various dye molecules is conducted, selecting the optimum dye for the displacement assay. The results collected demonstrate the rapid nature of the generated assay, specifying the optimized time span and amount of polymer powder required for analysis. A dose–response curve illustrates the limit of detection (LoD) of the methodology, building towards the analysis of spiked urine samples for amphetamine.

The research endeavors to highlight how a simple MIP-based assay can be developed towards the detection of an applicable real-world target, stressing the benefits of the assay over the more conventional means of analysis, while offering a low-cost alternative in the process. The commercial implications of this technology could be monumental, with the potential of the methodology being developed towards the analysis of a wide range of targets in many sectors.

## 2. Results

### 2.1. Analysis of Amphetamine MIPs

**Figure 2:**
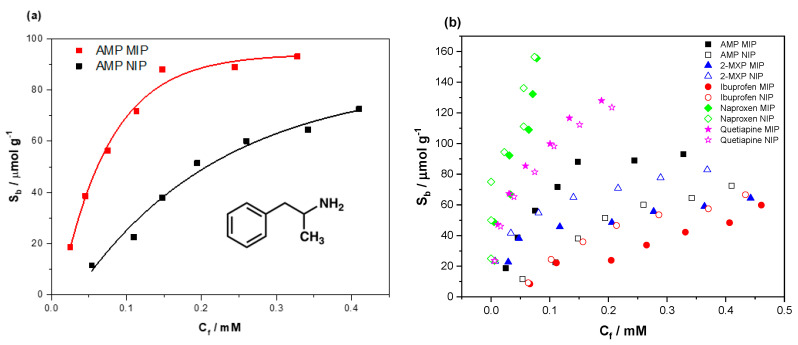
(**a**) The binding isotherm for MIP-32 (red) and nonimprinted polymer (NIP) (black) in response to increasing concentrations of (±) amphetamine (0.1–0.7 mM) in DI water, and (**b**) the binding isotherm for MIP-32 in response to compounds that stimulate false positives on current POC drug tests (ibuprofen, 2-MXP, pheniramine, bromopheniramine, naproxen and quetiapine fumarate).

To analyze the binding capabilities of the MIPs synthesized, batch rebinding experiments were undertaken. Known amounts of MIP/NIP powder (20 mg) were incubated with increasing concentrations of (±) amphetamine hydrochloride (0.1–0.7 mM) for 90 min before measuring the remaining free concentration of target in the solution. This was achieved by means of generating a calibration graph for amphetamine and extrapolating the λ_max_ (258 nm) absorbance values for the MIP/NIP solutions. Once the free concentration (C_f_, mM) remaining in the solution was confirmed, the amount of target bound to the MIP/NIP per gram (S_b_, μmol g^−1^) was calculated, allowing the construction of the corresponding binding isotherms. This was done for each of the MIP/NIP compositions, with the binding isotherm for the best-performing (highest binding capacity, specificity and imprint factor) MIP/NIP shown below (Figure 2a).

The data were fit using OriginPro8 (OriginLabs Corporation, Northampton, MA, USA) using an allometric (y = ax^b^) fit, modeling the binding isotherm of both the MIP (red line, R^2^ = 0.98419) and NIP (black line, R^2^ = 0.97139). MIP-32 had a maximum binding capacity of 93.13 μmol g^−1^ compared to 72.06 μmol g^−1^ of the NIP.

To evaluate and directly compare each composition, the imprint factors (IF) of each MIP/NIP composition was calculated, with the IF being defined as the amount of target bound by the MIP in comparison to the amount of target bound by the NIP at a given concentration. The concentration this calculation is performed at is low (C_f_ = 0.025 mM), giving an accurate representation of how the MIP/NIP composition initially performs under unsaturated binding conditions (see Supplementary Materials Table S1). MIP-32, the best-performing MIP, had a calculated IF value of 4.4 (at C_f_ = 0.025 mM).

The selectivity of the MIP towards (±) amphetamine was then scrutinized, incubating the MIP with compounds that cause current tests to generate false positives (Figure 2b). The compounds tested included ibuprofen (red circles), 2-MXP (blue triangles), naproxen (green diamonds) and quetiapine fumarate (pink stars). The data for the interaction of the compounds with both MIP and NIP are displayed, indicating whether the binding of each compound can be attributed to specific or nonspecific interactions. To facilitate a direct comparison of the binding, the data were allometrically fit (y = ax^b^) and the substrate bound (S_b_) was calculated for each compound for both corresponding MIP/NIP compositions at C_f_ = 0.025 mM (see Supplementary Materials Figure S1). The binding factor (BF) for each compound was then calculated in the same manner as the IF, generating a binding factor for each compound that could easily be compared (Table 1).

The affinity of 2-MXP, ibuprofen and naproxen towards the MIP was less than that of s the NIP, suggesting that the NIP offers greater binding modes towards these compounds than the MIP.

### 2.2. Preparation and Evaluation of the Dye Displacement Assay

**Figure 3:**
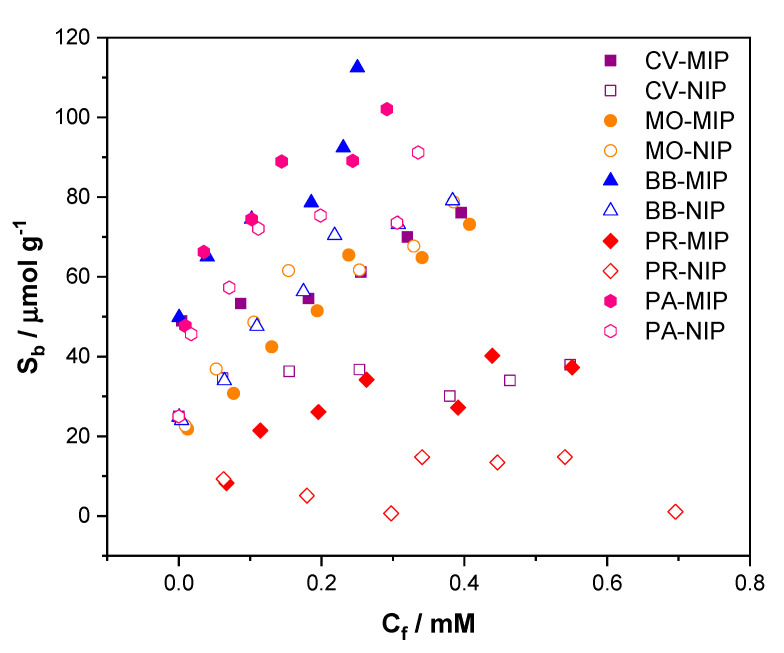
Binding isotherm for MIP-32 in response to varying concentrations of dye molecules (purple squares: crystal violet; orange circles: mordant orange; blue triangles: basic blue; pink hexagons: pararosaniline; red diamonds: phenol red).

Evaluation of the binding affinities of dye molecules was conducted in a similar manner to that previously described for the analysis of amphetamine and the analog compounds above. This time, crystal violet, basic blue, pararosaniline, mordant orange and phenol red had their binding capabilities assessed. The data were then fit, plotting the responsiveness of both the MIP and NIP, giving an indication of the compound with the greatest specific interaction towards the imprinted polymer (Figure 3). Of the dyes tested, crystal violet gave the greatest difference in binding between the MIP and NIP, with basic blue being a close second. Basic blue, pararosaniline, crystal violet and mordant orange demonstrated binding capacities with values of 113, 102, 76 and 73 μmol g^−1^, with phenol red having the weakest binding capacity of 37 μmol g^−1^.

To give a metric of how well each dye performed, the binding factor (BF) for each dye was calculated from the fitted data (Table 1). The BF was calculated at the same C_f_ (0.025 mM) as previously mentioned, allowing for a direct binding comparison. Crystal violet (1.7) provided the highest binding factor, with basic blue (1.6) also having a strong specific interaction towards the MIP. The other compounds proved to have either a greater interaction towards the NIP (phenol red, mordant orange) or an interaction of par between the reference and the MIP (pararosanaline).

**Figure 4:**
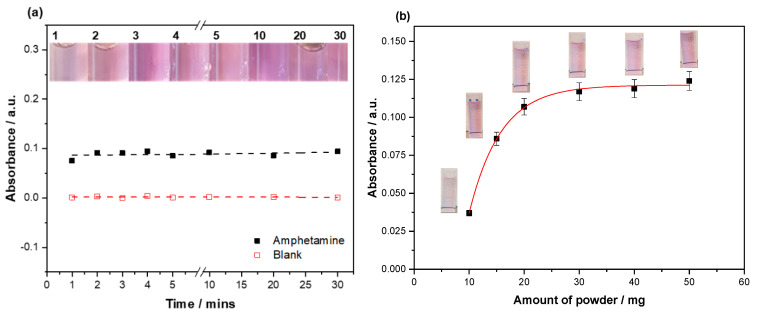
(**a**) The time dependency of the dye displacement when 20 mg of substrate displacement colorimetry (SDC) MIP powder was in the presence of (±) amphetamine hydrochloride (0.5 mg mL^−1^, black squares) and incubated with a blank (DI water, red squares); (**b**) effect the mass (10–50 mg) of the dye-loaded MIP has on the amount of dye displaced when incubated with (±) amphetamine hydrochloride (0.5 mg mL^−1^). The absorbances plotted are of crystal violet (λ_max_ = 590 nm).

Of the dyes tested, crystal violet was selected for incubation with the synthesized MIP. Crystal violet demonstrated the highest binding factor towards the MIP/NIP composition compared to the other dyes tested, demonstrating a high binding capacity alongside the naturally high extinction coefficient of the dye. Before a dose–response curve was conducted, the time for dye displacement to occur and the amount of SDC MIP powder required to perform the reaction were investigated. To this end, 20 mg of SDC MIP particles were incubated with aqueous (±) amphetamine hydrochloride (0.5 mg mL^-1^) and a blank (DI water) for 30 min, taking absorbance readings at defined intervals (Figure 4a). There was little to no change in the displacement of the dye after the one minute mark, with the data showing no correlation with the increasing time frame. This was further confirmed visually with a clear consistency in the shade of the dye displaced. The blank demonstrated no leaching of the dye, with the absorbance values remaining around the zero mark.

With the time for the displacement reaction to occur explored, scrutiny fell on the mass of SDC MIP and how it impacted the amount of dye displaced. This relationship was investigated by incubating varying masses of SDC MIP (5–50 mg) with aqueous (±) amphetamine hydrochloride (0.5 mg mL^−1^) and monitoring the absorbance of the dye displaced (Figure 4b). A clear difference in the amount of dye displaced from 5 to 30 mg can be seen. After this point, there was little change in the absorbance. This is also visually apparent, with the intensity of the violet color increasing up until 30 mg. The optimized mass was therefore carried forward to the dose–response, alongside the optimized time determined in the previous experiment.

### 2.3. Dye Displacement Assay Dose–Response

**Figure 5:**
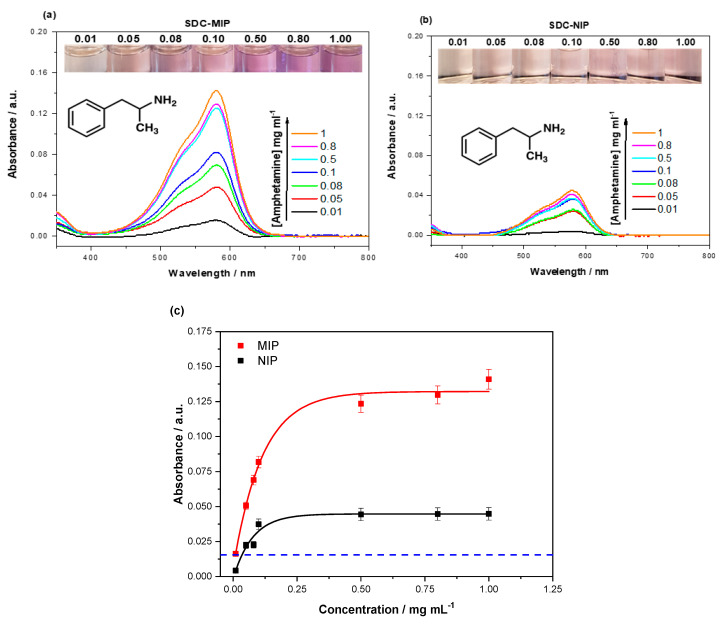
UV spectroscopy analysis of crystal violet (λ_max_ = 590 nm) in the filtrates of (**a**) SDC MIPs and (**b**) SDC NIPs (30 mg) that were incubated with varying concentrations (0.01–1 mg mL^−1^) of (±) amphetamine hydrochloride in water. (**c**) Comparison of the absorbance (λ_max_ = 590 nm) for the SDC MIP/NIP at the corresponding concentrations of amphetamine hydrochloride. Error bars are one standard deviation from the mean reading, and the blue dashed line is the limit of detection (LoD) (0.009 mg mL^−1^), as calculated by the 3σ rule.

To ensure the displacement assay performed as expected, the crystal-violet-loaded SDC MIPs were exposed to varying concentrations of (±) amphetamine (0.01–1 mg mL^−1^) and the amount of dye displaced was quantized with a UV spectrophotometer (Shimadzu-3600). The absorbance spectra were measured between 350 and 800 nm, capturing the λ_max_ (590 nm) of crystal violet (Figure 5a,b). The absorbance data were plotted and fit against the concentration of (±) amphetamine used using OriginPro8 (OriginLabs Corporation, Northampton, MA, United States) and an allometric (y = ax^b^) fit, modeling the displacement of dye from both the SDC MIP (red line, R^2^ = 0.98419) and SDC NIP (black line, R^2^ = 0.97139) (Figure 5c).

The dye-loaded MIP demonstrates a greater displacement than that of the NIP, with the amount of dye displaced beginning to plateau after 0.5 mg mL^−1^ amphetamine was introduced. The NIP plateaus a lot sooner with 0.1 mg mL^–1^, the greatest concentration to elicit an increased dye displacement. Therefore, the displacement from the MIP is observed to be quantitatively and qualitatively greater than that of the NIP, with the filtrate demonstrating a clear darker shade of purple.

### 2.4. Dye Displacement Selectivity Study

**Figure 6:**
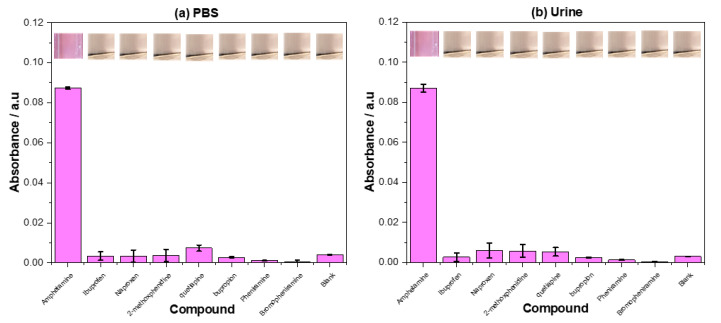
UV spectrophotometer analysis of the filtrates collected after exposing the dye displacement assay to (±) amphetamine, ibuprofen, naproxen, 2-methoxphenidine, quetiapine, bupropion, pheniramine, bromopheniramine (0.1 mg mL^−1^) and a blank in both. (**a**) PBS (1×) and (**b**) urine. Absorbances were recorded at λ_max_ = 590 nm. The absorbance of crystal violet displaced into the solution was reported.

The selectivity of the assay was analyzed in both PBS and spiked urine, analyzing (±) amphetamine and a host of compounds that stimulate false positives in current tests ((±) amphetamine, ibuprofen, naproxen, 2-methoxphenidine, quetiapine, bupropion, pheniramine, bromopheniramine). The filtrate from the analysis was visually analyzed and the absorbance readings were used to graph the data (Figure 6).

Of the compounds tested, amphetamine showed the greatest dye displacement when incubated with the assay, performing well in both PBS and urine. The standard deviation of the dye displaced in PBS was lower than that observed in urine, with the mean value of the dye displaced being practically identical. The other compounds tested in PBS showed little to no dye displacement in comparison to the amphetamine and the blank, with quetiapine demonstrating the greatest dye displacement of the compounds. The same was observed for the compounds in urine, though naproxen and 2-methoxphenidine showed a slightly increased crystal violet concentration in their filtrates. Comparing the PBS based measurements directly to the urine-based measurements, as a whole, the standard deviation was higher in the urine-based measurements with a small increase in the dye displaced by the competitive compounds.

## 3. Discussion

### 3.1. Evaluation of Amphetamine MIP Data

Compared to the other compositions tested, MIP-32 demonstrated the highest binding capacity (93.13 μmol g^−1^) in comparison to the corresponding NIP (72.06 μmol g^−1^). This fact meant the MIP had the highest calculated IF value of the MIPs analyzed, with an IF value of 4.4. As the IF is a direct numerical representation of the imprinting effect, it indicates that MIP-32 provided the greatest amount of specific binding sites towards (±) amphetamine in comparison to its corresponding reference NIP. This is not a surprising result, as the other MIP utilized acrylamide and styrene as functional monomers, providing little to no binding interactions with the amphetamine. Styrene had the potential to form π-stacking interactions, making it a plausible choice of monomer, though these interactions are much weaker than the ionic interactions seen between the methacrylic acid and the amine functionality present in the (±) amphetamine molecules. Racemic amphetamine was chosen as a template, as this draws parallels to the racemic amphetamine present in compounds such as Adderall. The MIP is imprinted towards the detection of both enantiomers, though the selectivity towards each individual was not tested. In a real-world scenario, it is likely that both versions of the compound would be present and is therefore of higher use to present a binding isotherm depicting the binding of the racemic mixture rather than the individual enantiomers.

Of the compounds in determining the selectivity of the MIP, quetiapine was the only compound that demonstrated higher binding towards the MIP than the NIP. This was reflected in the IF values calculated for each of the compounds, with quetiapine having an IF value of 1.13. The other compounds gave IF values below 1, indicating the nonspecific interactions towards the NIP were stronger than the specific interactions towards the MIP. This is a peculiar result. When comparing the structures of the compounds tested against those of amphetamine (Supplementary Materials Figure S2), quetiapine is one of the least similar structures. The explanation of this binding may lie in the number of heteroatoms present in the molecule, which offers multiple methods of binding to the MIP. The “tail” of the molecule may have aided binding, providing easy modes of rotation within the structure and a plausible method of attaching to the surface of the MIP/NIP composition rather than the imprinted nanocavities. Another explanation for the higher affinity of the compounds towards the NIP than MIP lies in the functionalized space available for the compound to bind to the surface of the polymer. The MIP contains nanocavities tailored towards the template molecule, covering the surface with complementary amphetamine-shaped cavities. These cavities may in fact oppose the binding of other molecules, inhibiting or weakening the binding forces that would normally be available. It stands to reason that the NIP offers greater available space for potential binding as it is not littered with complementary binding sites engineered towards amphetamine and can therefore provide a larger amount of nonspecific interactions. Baggiani et al. demonstrated this in their work, where they demonstrate that a NIP has to provide a large amount of binding towards molecules for the MIP to also exhibit the same trait [40]. Furthermore, the porosity of the MIP has shown to increase the binding of a target molecule, with an increased surface area and a number of binding sites [41], relating back to the amount of nonspecific binding sites and potential areas where other molecules other than the template molecule cannot bind.

The hypothesis that most of this higher affinity to analog species can be attributed to nonspecific binding was further confirmed in displacement studies showing that these analogs do not decrease the selectivity of the resulting assay. This can be explained by the fact that dye molecules binding to nonspecific areas of the MIP/NIP composition are easily washed off. Only dye molecules that specifically interact with the binding sites inside the nanocavities remain bound. The target will displace them from these binding sites, whereas the analogs that do not interact with these specific binding sites do not displace the dye molecules.

### 3.2. Evaluation of Dye Binding

Though the dyes tested primarily fell within a tricyclic structure (bar mordant orange), they all interacted differently towards the MIP (Supplementary Materials Figure S2). Crystal violet proved to be the dye that provided the most specific interaction towards the MIP, followed closely by basic blue. This is unexpected as crystal violet contained multiple tertiary amines, whereas pararosaniline contained multiple primary amines and is closer to the functionalities present in amphetamine. Basic blue also contained multiple tertiary amine functionalities, though with longer N-chain lengths than the methyl groups present in crystal violet. The longer chain lengths would be a plausible reason for the diminished binding capabilities, though pararosaniline would be expected to provide the highest binding based on structure alone. The relative size of the molecules also has an effect on the perceived binding, though basic blue has a much larger structure, followed by crystal violet and finally pararosaniline, which are similar in size to amphetamine. Mordant orange and phenol red provide no specific interaction to the MIP, though this can be attributed to their lack of amine functionality. This speculation is strengthened by the research conducted by Dorko et al., who characterized the interactions between different amine architectures and a MIP [42]. Many factors (including structure and size) affect the binding of a similar molecule to the MIP; however, most importantly, different factors affect the binding of a compound in different ways. Crystal violet therefore is the dye that was brought forward for the dye displacement assay, demonstrating the highest BF and a reasonable binding capacity towards the MIP.

### 3.3. Effects on Incubation Time and Mass of SDC MIP

The amount of the dye displaced by amphetamine was not seen to increase past the period of one minute, which could be due to the difference in the binding factor between the dye molecule and amphetamine. It was shown in a previous study (Lowdon et al. [39]) that the difference in the binding affinities of the molecules was one of the driving forces behind the displacement reaction. It has previously been reported that the interaction was not as fast as it has been observed here; however, the difference in the binding factors in this scenario is much greater than previously reported. The rapidness of the displacement can therefore be attributed to this large difference in binding affinities towards the MIP. This difference in binding affinities might be derived from the differences in size between crystal violet and amphetamine, with crystal violets affinity being dependent on the highly accessible amine functionalities on the edges of the molecule. As there is no perceivable advantage for longer incubation periods, the lower incubation period was brought forward, strengthening the argument for the displacement assay being a fast method of analysis and making the assay commercially interesting. The analysis of the blank demonstrated absorbance values of/or around zero, indicating that there was no visible or quantifiable leaching of the dye in solution over the time range analyzed. Again, this is good as it means that the sensor is robust over a longer analysis period.

Optimization of the amount of dye-loaded MIP required for analysis revealed that 30 mg was the preferable amount, with the amount dye displaced above the amount only fractionally increased. Below this amount, the dye displaced was greatly reduced and consequently impeded the detection of low concentrations of amphetamine. As the concentration of amphetamine was relatively high (0.5 mg mL^–1^), it made the most sense to select a higher mass of the MIP powder, maximizing the possibility of low concentration displacing larger quantities of dye. A saturation effect is observed about the 30 mg, indicating that there is a point where the amount of the MIP becomes less of a limiting factor than the concentration of the amphetamine presented for incubation.

Considering these factors, the overall preparation time of analysis was just a few minutes, including the weighing of the SDC powder, incubation of the sample and filtration of the mixture. This time would be decreased in a real-world setting with the amount of SDC MIP powder already weighed, and a solution could be engineered towards the rapid filtration of the mixture. When compared to other methods of analysis this is still very fast, with other rapid methods taking around 5 min (longer for more sensitive methods such as HPLC/mass spectroscopy) [43,44]. This is a distinct commercial benefit as it shows that the MIP-based assay can compete in a field where the rapidness of analysis is essential.

### 3.4. Dye Displacement Dose–Response Evaluation

The introduction of increasing concentrations of (±) amphetamine hydrochloride (0.01–1 mg mL^−1^) to both dye-loaded MIP and NIP yielded dye displacement. The displaced dye for the NIP can be related to the nonspecifically bound dye displaced by the amphetamine nonspecifically interacting with the polymer. The displacement of dye from the MIP was much greater than the displacement from the NIP, implying that the dye occupied complimentary nanocavities within the structure, strengthening the argument for the imprinting effect playing a role in the displacement of the dye as it shares some affinity with the amphetamine-specific binding sites. As previously discussed, this may be due to the easily accessible nature of the amine functionalities in the structure of the crystal violet dye being able to bind to the methacrylic acid functionalities inside the nanocavities.

The linear range of the dye displaced from the MIP lies between 0.01 and 0.20 mg mL^−1^, with concentrations higher than this showing a diminished capability of dye displacement. Though the linear range is not vast, it supports the analysis of samples at lower concentrations that are more applicable to physiological concentrations. Therefore, the lowest observable dye displacement occurs at an amphetamine concentration of 57 μM. The LoD was calculated using the 3σ rule, three times the maximum standard deviation within the measured samples (0.009 mg mL^−1^). This rule does not take into consideration the dye displaced by the NIP, which means the LoD falls within the realms of nonspecific dye displacement. Typical urine concentrations are in the same order of magnitude within the first day upon intake but rapidly fall off in the next day to concentrations that are 100–1000 times lower than the operating regime of the sensor [45]. This indicates that the system still needs to be optimized for regulatory drug abuse testing but still has a lot of commercial potential in the routine screening of unknown solid powder samples. The assay could easily be used in a semi-quantitative capacity for this application, with the intensity of the color change being a clear reflection of the amount of target present without the need for a UV spectrophotometer to confirm.

### 3.5. Selectivity of the Displacement Assay

Scrutiny drawn towards the selectivity of the assay highlighted the capability of the sensing method to selectivity bind amphetamine in both PBS and urine. The selectively was confirmed both visually (purple filtrate) and by UV spectrophotometer, allowing a metric to be related to each of the compounds tested. As observed in the selectivity tests of the raw MIP, the displacement assay demonstrated a low level of dye displacement towards the presence of quetiapine. This can be attributed to the structural feature previously discussed, enabling the quetiapine to provide a low level of dye displacement. This was seen to be accentuated in PBS, though the displacement was barely greater than that of the blank, with it only being noticeable in the metric and not under visual observation. Of the other compounds tested, none demonstrated a displacement much greater (0.005 a.u) than that of the blank, and all paled in comparison to the dye displacement facilitated by the amphetamine. The minor displacement of the dye could prove problematic in the analysis of lower concentrations of amphetamine, though for a rapid on the stop measurement, the benefits outweigh the cons. This does not reflect badly on the calculated limit of detection, as the value quoted (0.009 mg mL^−1^) falls above the displaced amount of dye. This is a promising result considering that these compounds are common false positives in current tests, and the assay seems to have overcome this issue. The more rigid nature of the polymeric receptors may be the causality of the higher degree of selectivity, hindering the binding of larger molecules. This has huge commercial potential for the assay and it demonstrates the selective nature of the MIPs and how this can be utilized in the displacement assay to produce a result that would normally be impossible in other test formats.

## 4. Materials and Methods

### 4.1. Chemicals and Reagents

Prior to polymerization, stabilizers were removed from the functional and crosslinking monomers by passing the reagents over a column packed with alumina. All chemicals and solvents were obtained from Sigma-Aldrich (Zwijndrecht, The Netherlands). All tested analyte solutions were prepared with deionized water with a resistivity of 18.2 MΩ cm^–1^ or with phosphate-buffered saline (PBS) solutions unless stated otherwise (license reference 506207WCO, regarding the handling and storage of Class 1 compounds within The Netherlands).

### 4.2. Synthesis of Molecularly Imprinted Polymers

The procedure for the synthesis of the optimized MIP followed the method previously reported [39]. In essence, a mixture of the functional monomer methacrylic acid (MAA, 0.27 mL, 3.1 mmol), functional crosslinker ethylene glycol dimethacrylate (EGDMA, 1.73 mL, 9.2 mmol) and thermal initiator azobisisobutyronitril (AIBN, 50 mg, 0.30 mmol) in 3 mL of dimethyl sulfoxide (DMSO), together with (±) amphetamine hydrochloride (50 mg, 0.29 mmol), was prepared in a 15 mL glass vial. The mixture was degassed with N_2_ before initiating the polymerization reaction. The vial containing the reaction mixture was then placed into an oil bath and heated at 65 °C for 12 h, allowing for the full completion of the polymerization reaction. The MIP was milled several times using a Fritsch Planetary Micro Mill Pulverisette 7 Premium Line (700 rpm, 3 min, 10 mm balls). After milling, the particles were sieved at a 1.0 mm amplitude using a Fritsch Analysette 3 for 2 h or until a sufficient amount of the polymer was on the collection plate to achieve microparticles with sizes smaller than 100 µm. Finally, the template molecule was removed from the MIP powders by continuous Soxhlet extraction with a 1:10 mixture of acetic acid and ethanol for 12 h, followed by further extraction with pure ethanol for a further 12 h and drying of the particles at 65 °C overnight. A reference non-imprinted polymer (NIP) was also prepared in parallel. All MIP composition tests can be found in Supplementary Materials Table S1, alongside their corresponding imprint factor (IF) values.

### 4.3. Optical Binding Experiments

Optical batch rebinding experiments were evaluated with a Shimadzu UV-3600 spectrophotometer. A dilution series (0–0.7 mM) was prepared by varying aliquots of a 1 mM stock solution of amphetamine hydrochloride added to the PBS solution. Subsequently, 20 mg of MIP/NIP powder was added to 5 mL of each concentration. The resulting suspensions were placed on an orbital shaker (125 rpm) for 1 h at room temperature. After filtration, the free concentration of substrate in the filtrate was determined by UV–vis spectroscopy, allowing the construction of the corresponding binding isotherm. As a measure of specificity, and in order to compare the different MIP compositions, the imprint factor (IF) was determined at C_f_ = 0.025 mM. This method was repeated for each of the MIPs synthesized in this study, alongside a selectivity study where an array of related compounds (ibuprofen, 2-methoxphenidine, naproxen, quetiapine, 2-methoxphenidine) binding towards the MIP was tested. Alongside the aforementioned analogs tested, a series of dye molecules (crystal violet, basic blue, pararosaniline, mordant orange and phenol red) had their affinities towards the MIP and NIP tested, identifying the most feasible dye for SDC applications.

### 4.4. Preparation of Substrate Displacement Colorimetry MIP Particles

To 50 mL of aqueous crystal violet (1 mM) was added 1 g of MIP powder. This was then left to stir (150 rpm) at room temperature for 1 h, allowing the dye molecule to bind to the MIP. The resulting suspension was then collected by means of filtration and washed rigorously with DI water (3 L) until the filtrate ran colorless. To ensure there was no leaching of unwanted dye from the MIP, a Shimadzu UV-3600 spectrophotometer (Shimadzu, Kyoto, Japan) measured the UV absorbance of the filtrate collected. The washed-dye-loaded MIP particles were dried in an oven at 80 °C overnight, yielding the dried SDC MIP powder ready for analysis. The same process was completed for the NIP, providing a reference material for comparison in future experiments.

### 4.5. Time Dependency of Dye Displacement

SDC MIP particles (20 mg) were incubated with 5 mL of (±) amphetamine hydrochloride (0.5 mg mL^–1^) in PBS (1×), evaluating the amount of dye displaced by means of UV spectroscopy (λ_max_ = 590 nm) at defined time intervals (1, 2, 3, 4, 5, 10, 20 and 30 min). Each time interval was analyzed individually with the SDC MIP particles removed at the specified time interval stated. The filtrate was then analyzed with a Shimadzu-3600 UV spectrophotometer (3.5 mL quartz cuvette, path length of 1 cm) and the maximum absorbance (λ_max_ = 590 nm) noted. Each time interval was repeated in triplicate, with the mean absorbance reported.

### 4.6. Optimization of the Mass of SDC Particles for Analysis

(±) Amphetamine hydrochloride in PBS (1×) (10 mL, 0.5 mg mL^–1^) was incubated with various masses of SDC MIP particles (10, 15, 20, 30 and 40 mg) for 1 min before the filtration of the sample. The remaining solution was then collected and the absorbance of the crystal violet (λ_max_ = 590 nm) assessed with a Shimadzu-3600 UV spectrophotometer (3.5 mL quartz cuvette, path length of 1 cm). Each mass of SDC particles in the hydrogel mesh was repeated in triplicate, with the average absorbance and standard deviation reported.

### 4.7. SDC Dose–Response

A dose–response study was constructed by incubating the synthesized SDC MIP (30 mg) with varying concentrations of (±) amphetamine hydrochloride (0.01–1 mg mL^–1^) in PBS (1×) for 1 min before the filtration of the sample. The filtrate proceeded to be analyzed by a Shimadzu-3600 UV spectrophotometer (3.5 mL quartz cuvette, path length of 1 cm) collecting the spectrum for each sample (350–800 nm). The maximum absorbance of each concentration (λ_max_ = 590 nm) was then plotted against the concentration of (±) amphetamine hydrochloride added, allowing graphing of the dose–response curve. The process was then repeated for the NIP, providing a reference for the displacement reaction to be compared against.

### 4.8. Selectivity of the Dye Displacement

To study the selectivity of the displacement reaction, the SDC MIP was exposed to 5 mL of various targets (0.1 mg mL^−1^) (ibuprofen, 2-methoxphenidine, naproxen, quetiapine, 2-methoxphenidine) in PBS (1×) before visually assessing the filtrate and collecting the absorbance spectrum. The process was then repeated for the analysis of each of the compounds in urine (collected from one of the authors, and spiked with each of the specified compounds), enabling a comparison of the assay in a simulated real-world sample. Each analysis was repeated in triplicate with the average values and standard deviation reported.

## 5. Conclusions

Overall, the research highlights how a MIP can be converted into a dye displacement assay that offers huge potential for the rapid analysis of compounds. The assay was shown to work in a model environment (PBS) and a biofluid (urine), demonstrating the assay’s versatility. The selectivity of the assay is superior in comparison to other commercial detection platforms but is not sensitive enough yet for routine drug testing. This issue can be overcome by employing a more sophisticated method of producing MIPs in the future. At this point in the research, we wanted to illustrate the commercial potential of the assay and relied upon bulk polymerization as it proved cheap, fast and scalable. A more advanced polymerization protocol, creating MIPs with high binding affinities, in combination with tailor-made dyes with high extinction coefficients, could enhance the sensitivity of the assay by multiple orders of magnitude. On the other hand, the current research exemplifies the use of this assay in a laboratory environment for the detection of amphetamine in, e.g., unknown powder samples, seized by authorities. This powder would only have to contain a trace amount of amphetamine for the assay to give a positive signal. This finding, in combination with its fast processing time, ease-of-use, superior selectivity and low cost, indicates that the assay in its current form already has significant commercial potential. In addition, with the optimization of the sensitivity and widening of the application area to the verification of other targets with limited selectivity, the commercial impact could become even bigger in the coming years.

## Figures and Tables

**Table 1:** The amount of each compound bound to both MIP and NIP (S_b_) at C_f_ = 0.25 mM, and their corresponding binding factors (BF).

Compound	S_b_ (μmol g^−1^) (C_f_ = 0.025 mM)	Binding Factor (BF)	Dye	S_b_ (μmol g^−1^) (C_f_ = 0.025 mM)	Binding Factor (BF)
(±) amphetamine	MIP: 18.90 NIP: 4.29	4.4	crystal violet (CV)	MIP: 48.43 NIP: 29.29	1.7
2-MXP	MIP: 27.67 NIP: 38.30	0.71	mordant orange (MO)	MIP: 27.38 NIP: 32.17	0.85
ibuprofen	MIP: 5.77 NIP: 13.91	0.41	basic blue (BB)	MIP: 61.82 NIP: 37.86	1.6
naproxen	MIP: 73.33 NIP: 96.47	0.76	phenol red (PR)	MIP: 5.89 NIP: 9.68	0.6
quetiapine	MIP: 59.57 NIP: 52.69	1.13	pararosaniline (PA)	MIP: 63.26 NIP: 57.52	1.1

## Supplementary Materials

The following are available online. Figure S1: Plotted selectivity isotherm for MIP-32, Figure S2: The chemical structures of potential chemical interfaces and dye molecules, Table S1: Chemical composition of the tested MIPs and their corresponding imprinting factors.
