# Supplementary material for: A Molecularly Imprinted Polymer-based Dye Displacement Assay for the Rapid Visual Detection of Amphetamine in Urine
Source: Molecules. 2020 Nov 10;25(22):5222. doi: 10.3390/molecules25225222 (PMC7696774; doi:10.3390/molecules25225222)
Supplement: Supplementary file 1 [file molecules-25-05222-s001.pdf]

*** Supplementary Information ***

A Molecularly Imprinted Polymer Based Dye Displacement Assay for the Rapid Visual Detection of Amphetamine in Urine

Joseph W. Lowdon*[1], Kasper Eersels[1], Rocio Arreguin-Campos[1], Manlio Caldara[1], Benjamin Heidt[1], Renato Rogosic[1], Kathia Jimenez Monroy[1], Thomas J. Cleij[1], Hanne Diliën[1], Bart van Grinsven[1]

[1] Sensor Engineering Group, Faculty of Science and Engineering, Maastricht University, PO Box 616, 6200 MD, Maastricht, the Netherlands

* Correspondence: joe.lowdon@maastrichtuniversity.nl

Supplementary Table S1: Chemical compositions of the MIPs tested with their associated imprinting factor (IF) towards amphetamine at C_f = 0.025 mM.

MIP	MAA (mmoles)	AA (mmoles)	Styrene (mmoles)	EGDMA (mmoles)	DMSO (mmoles)	AIBN (mmoles)	Amphetamine Hydrochloride (mmoles)	IF (C_f = 0.025 mM)
**31**	3.1	-	-	9,2	3	0.3	0.29	0.99
**32**	-	3.1	-	9.2	3	0.3	0.29	4.4
**33**	-	-	3,1	9.2	3	0.3	0.29	0.74

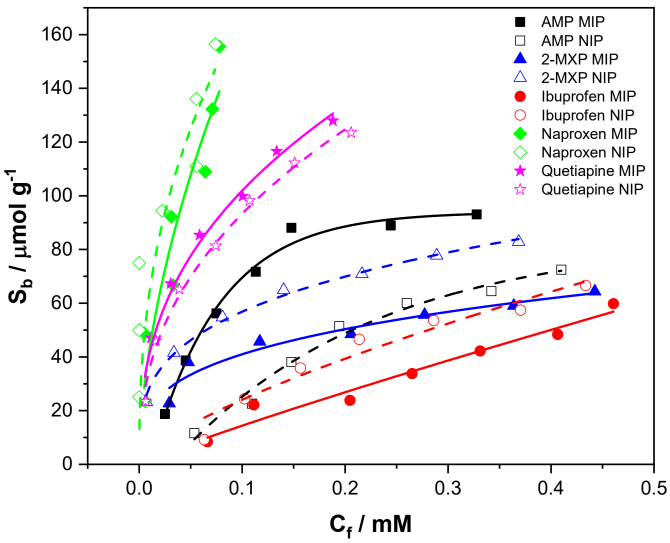

Supplementary Figure S1. The fitted selectivity binding isotherm for MIP-32, demonstrating the binding of amphetamine (black squares), 2-MXP (blue triangles), ibuprofen (red circles), naproxen (green diamonds), and quetiapine (pink stars).

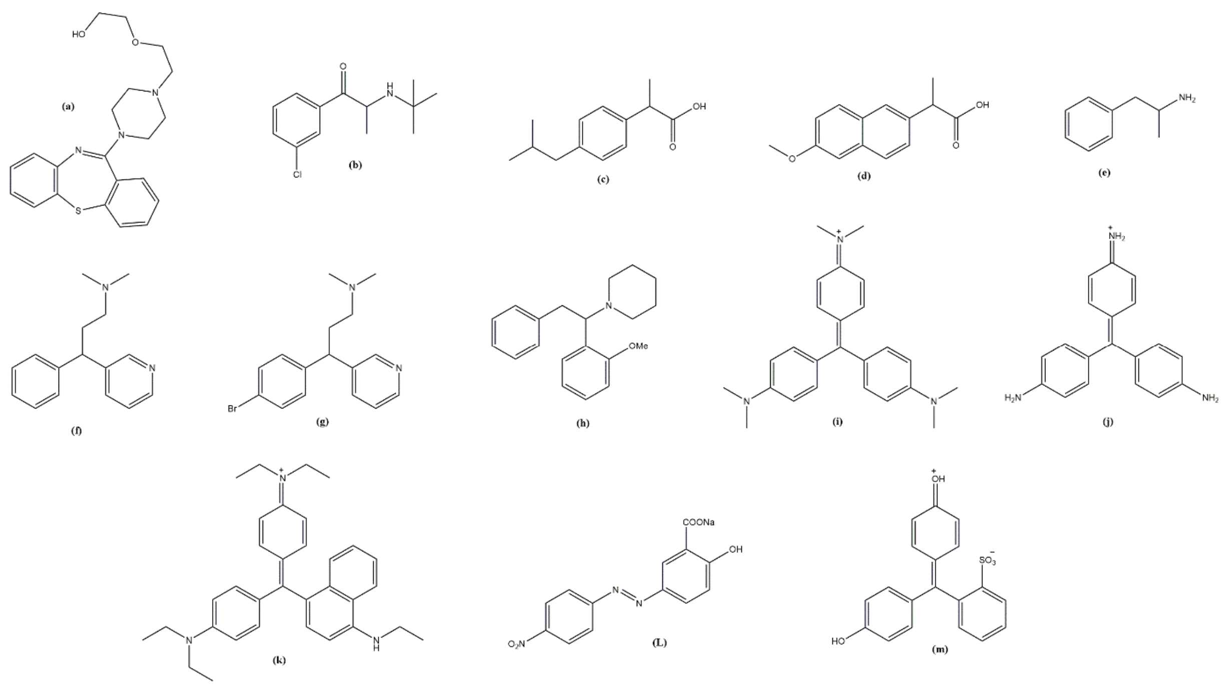

Supplementary Figure S2. The chemical structures of (a) quetiapine, (b) bupropion,(c) ibuprofen, (d) naproxen, (e) amphetamine, (f) pheniramine, (g) bromopheniramine, (h) 2-methoxphenidine, (i) Crystal violet, (j) pararosaniline, (k) basic blue, (l) mordant orange, and (m) phenol red.
